# An integrated analysis of micro- and macro-habitat features as a tool to detect weather-driven constraints: A case study with cavity nesters

**DOI:** 10.1371/journal.pone.0174090

**Published:** 2017-03-20

**Authors:** D. Campobello, J. Lindström, R. Di Maggio, M. Sarà

**Affiliations:** 1 Section of Animal Biology, Dept. STEBICEF, Università di Palermo, Palermo, Italy; 2 Institute of Biodiversity, Animal Health and Comparative Medicine, University of Glasgow, Glasgow, United Kingdom; Hungarian Academy of Sciences, HUNGARY

## Abstract

The effects of climate change on animal populations may be shaped by habitat characteristics at both micro- and macro-habitat level, however, empirical studies integrating these two scales of observation are lacking. As analyses of the effects of climate change commonly rely on data from a much larger scale than the microhabitat level organisms are affected at, this mismatch risks hampering progress in developing understanding of the details of the ecological and evolutionary responses of organisms and, ultimately, effective actions to preserve their populations. Cavity nesters, often with a conservation status of concern, are an ideal model because the cavity is a microenvironment potentially different from the macroenvironment but nonetheless inevitably interacting with it. The lesser kestrel (*Falco naumanni*) is a cavity nester which was until recently classified by as Vulnerable species. Since 2004, for nine years, we collected detailed biotic and abiotic data at both micro- and macro-scales of observation in a kestrel population breeding in the Gela Plain (Italy), a Mediterranean area where high temperatures may reach lethal values for the nest content. We show that macroclimatic features needed to be integrated with both abiotic and biotic factors recorded at a microscale before reliably predicting nest temperatures. Among the nest types used by lesser kestrels, we detected a preferential occupation of the cooler nest types, roof tiles, by early breeders whereas, paradoxically, late breeders nesting with hotter temperatures occupied the overheated nest holes. Not consistent with such a suggested nest selection, the coolest nest type did not host a higher reproductive success than the overheated nests. We discussed our findings in the light of cavity temperatures and nest types deployed within conservation actions assessed by integrating selected factors at different observation scales.

## Introduction

A growing body of literature demonstrates the effects of human-induced global warming on the structure and distribution of populations and communities across taxa [1, 2, 3]. One obstacle in developing a better understanding of these changes is that while organisms typically seek for suitable local conditions (microhabitats), species distribution models (SDMs) usually rely on climate data from much larger scales [4, 5]. Global warming effects and their interactions with other stressors have been typically examined either at a macro- or micro-scale, and an increasing number of studies have been pointing to the urgency of exploring the mechanisms and dynamics of the combination between factors at different scales of observation [6, 7]. Recently, Varner and Dearing [5] showed that there is a substantial temperature difference between the coarse measurements from weather stations, used for SDMs, and those recorded in the micro-habitat, which are, in fact, experienced by individuals of the American pika (*Ochotona princeps)*. Consequently, areas assessed as unsuitable by classic and correlative Species Distribution Models turned out to be optimal refugia in a higher-resolution analysis [8, 9].

Isolated analyses at either scale of resolution do not contribute to improving conservation measures or detecting ecological constraints for vulnerable species. Analyses of broad-scale trends of climate change do not reflect the real vulnerability of organisms to multiple climatic and nonclimatic stressors to which they are exposed in their smaller spatiotemporal scale [2]. Varner and Dearing [5] included both macro- and micro-habitat features in their analyses, however, they did not address the potential cumulative or interactive effects between factors at these two different scales of observation. Here, we use data on cavity-nesting birds, an excellent model for investigating such potential interactive effects of biotic and abiotic conditions recorded in their both macro- (i.e. breeding site) and micro- (i.e. hole-nests) habitat.

Nest temperatures directly or indirectly affect all life-history stages of avian reproduction [10, 11]. Contrary to temperate areas where eggs and nestlings are threaten by lethal low temperatures [12, 13], in warmer breeding regions, thermal stress induced by extreme high temperatures is a real risk to hatching success [14] and chick growth [15, 16]. Internal physiology of the chicken egg, for example, changes drastically with temperatures above 38.9°C which is considered as a maximum threshold for egg survival [17].

Nest temperatures are affected by myriads of factors although we know little about their combined effect. Ambient temperature is intuitively the main factor affecting nest conditions and, in fact, its trend is shown together with nest temperature curves for a visual comparison (e.g. [18, 19, 20]). Despite its important role, macroenvironment temperature has not been added yet as a term in the predicting models to quantify its contribution in the nest temperatures. In a few cases [21], the difference between ambient and nest temperature has been quantified but we still lack of a model including not only the ambient temperature but also other biotic and abiotic factors revealed important determinants of nest temperatures [18, 19, 15, 20]. Among these, the type of cavity is an important predictor of nest temperatures as they may have different types insulation. As a few examples, tree features are the main temperature predictors inside nests of Northern flickers (*Colaptes auratus*), although they do not affect flicker reproductive performance [18]. Nest boxes used by rollers (*Coracias garrulus*) were colder than natural cavities [20] but hotter in the tropics when used by an arboreal marsupial, the mahogany glider (*Petarus glaciris*) [19].

Excessive heat inside potential nest sites is therefore a negative condition threatening successful breeding attempts and to be ideally avoided by breeders [14]. In addition to nest characteristics, behavioral traits, such as nest site selection, may represent a valid measure to adopt for mitigating adverse nest conditions when suitable microenviromental features are available [10, 11]. Selection of optimal nest sites are expected to be under strong selection pressure [22, 23, 24]. However, high-quality resources are often limited in nature. Since the first description of Ideal Free Distribution theories, there is substantial evidence showing that, when there is quality variation in resources, the best are taken first and poorer ones are used only successively [25, 26].

In this study we, i) verified whether selected biotic and abiotic factors were able to modify the temperatures recorded at a macro-scale (i.e. by weather stations) into different values recorded at a micro-scale inside the nest (i.e. by thermo-loggers). Then, due to Mediterranean climate characterized by high summer temperatures, we ii) hypothesized a higher occupancy of the cooler nest type and, finally, iii) examined whether the factors able to influence the nest temperatures were also able to affect kestrel reproductive parameters, such as egg hatching and nestling fledging.

We conducted this study, in a population of a cavity nester, the lesser kestrel (*Falco naumanni*), breeding colonially in the Gela Plain (Sicily, Italy). Kestrel reproductive parameters and population size as a whole are influenced by both macro- and micro-scale environmental factors, such as rainfall or nest orientation [27, 28, 15], although we still do not know to what extent the effects of the two scales are correlated locally and whether individuals respond to potential thermal variation between nesting sites. In addition to providing an easily quantifiable microhabitat (nest cavities), lesser kestrels in our study site provide an exceptional model to address questions on the quality and opportunity of conservation measures. As lesser kestrels are still on the verge of status concern [28, 29] and because a sudden decrease of local nest sites, in the effort of providing more breeding opportunities, we have in fact recently deployed artificial nests, hence made available an opportunity to compare the efficiency of this common conservation measure with nesting natural conditions.

## Methods

### Study species and area

Lesser kestrels are small migratory raptors that breed in the Mediterranean area in open and dry cereal steppes [27, 28]. It is a facultative colonial species that breeds in mono- or multi-species assemblages [30, 31]. It is a monogamous species [32, 33] with biparental incubation and nestling provisioning [27]. Since 2004, we have been monitoring the largest Sicilian population of lesser kestrels nesting in the Gela Plain (Sicily, 37°07'N, 14°19'E; [28, 34], an agricultural landscape extended over 450 km^2^ with a mosaic of pseudo-steppes [35]. As in most avian species [10, 11], seasonality affects kestrel reproductive outcome, with late kestrel breeders being less successful than the early ones (unpublished results). Several farmhouses and rural buildings are scattered across the plain with each hosting one kestrel colony. There are 35 colonies in our study area and the average distance between them is 7 ± 0.5 km (mean ± SE, range: 0.6–19 km, [36]).

Lesser kestrels are secondary-cavity nesters and therefore find in these buildings numerous cavity nests, such as holes in walls and under saddle-shaped tiles [34, 37]. In most colony buildings, rooftops have been collapsing gradually with time as they are abandoned by people since 1950's. We have been placing 44 wooden nest-boxes of standard size and shapes [38] since 2010, both on existing colony buildings and in new sites. Nest boxes were made of 20-mm-thick pine wood. The internal size was 25 cm height, 25 cm width, and 50 cm depth. The entrance was 6 cm of diameter opened in the middle of the front wall. Venting was 1.5 cm height and placed all along the four walls below the box ceiling.

### Measurements of breeding and temperature parameters

We conducted our investigation across a period of nine years, April-July 2004-2013, except in 2008 (hereafter, for brevity 2004-2013) for a total of 1,157 nests monitored (where at least one egg was laid) across the 35 colonies (S1 Table, S1 and S2 Data). Each nest was checked 3-6 times per season so that, for each breeding pair, we could record the type (Box, Hole, Tile), characteristics (volume, cm^3^) and orientation of nests and the first laying day (Laying Day, hereafter) expressed as Julian day (January 1^st^ = 1). Volume was quantified by assuming the nest cavity shaped as a rectangular parallelepiped. Length was measured from the lower rim of the entrance to the bottom of the hole, width was the distance between the two midpoints of the lateral sides of nest entrances, and finally height as the distance from the two midpoints of bottom and top sides of nest entrance. Nest orientation was classified as Cold if the nest was facing W, NW, N, NE sides, whereas nests facing E, SE, S, SW were classified as Hot. We quantified nest content, including number of eggs, nestlings and adults found inside the nests and nestling age [34]. Specifically for this investigation purposes, we recorded the number of nestlings and their age as this may have influenced the nest temperature. Each class of nestling age corresponds to 7 days and was determined from nestling size and contour feather growth. Specifically, we identified four ages (A-D) characterized by the following features: A, from chicks coated with first down not standing up and shut eyes to chicks with first down, standing up and open eyes; B, first down and emerged primary pins; C, first down in regression and outer primaries emerged ≤ 1/3 of their length; D, from large remains of first down still on head and body and outer primaries emerged > 1/3 of length to first down absent or little traces on head and mantle, outer primaries completely emerged. As part of a long-term investigation [28, 39], we banded all individuals found inside the nest with numbered aluminum and plastic colored bands for remote identification.

The SIAS (Servizio Informativo Agrometereologico Siciliano) is a regional network of 96 automatic stations that record and store temperature, relative humidity, rain precipitation and wind speed data every hour. We used one of their stations located at 70 m a. s. l. in the proximity of the Gela town (4112703N; 440838E) and approximately 2-8 km from the kestrel colonies to obtain hourly temperature records over the entire kestrel breeding period. In the last four years of our study period, 2010-2013, across 11 colonies, we positioned 100 thermologgers inside 108 nests (23 in 2010, 23 in 2011, 25 in 2012, 37 in 2013) of which 10 were in nest-boxes, 46 inside wall holes and 52 under roof tiles. Eight nests failed early in the season and therefore we still had time to relocate those thermologgers in other eight nests. Thermologgers were HOBO U12-012 (Onset Computer Corporation, Bourne, MA, USA, recording temperatures from -20° to 70°C with an accuracy ± 0.35°C from 0° to 50°C) that recorded T°C every hour. To prevent their dislocation by breeders, we attached each thermologger to the nest wall or ceiling by using silicone caulk with a gluing gun. Although nests differed in types and volumes, thermologgers were always placed within 20 cm from the clutch. In 74% of the total nest thermologgers were recording since the laying day of the first egg whereas in the rest of the nests temperature recording started from 1 to 34 days later than the laying day of the first egg. This is because it was not always possible to predict the exact location of each nest beforehand. In all cases, thermologgers were in place and recording nest temperatures at the time of the first egg hatching until chick fledging or nesting failure. Once removed from the nest, thermologger data were downloaded via USB cable and HOBO software (version 3.7.0).

### Data analysis

#### Abiotic and biotic factors predicting nest temperatures

We used linear mixed models (e.g. [40]) to investigate the role of biotic and abiotic factors recorded at different scales of observations to predict nest temperatures. When fitting different models, we used Gaussian distributions as nest temperature was our response continuous variable. To compare competing models and select the most parsimonious ones, we used the Akaike’s Information Criterion (AIC, e.g. [41]) and Likelihood Ratio Testing (LRT). For the purposes of model selection, the candidate models, including all main effects and their two-way interactions, were fitted using maximum likelihood with Laplace approximation. We also validated the final models by checking the assumption of normality of residual errors in the case of normal error distribution. We conducted the analyses in R 3.0.1 [42] (R Core Team 2013) with the R package lme4 [43]. Prior to the following analysis, we checked for collinearity between potential predictors as assessed by Variance Inflation Factor (VIF) and excluded those with VIF values larger than two [40]. Specifically, we examined the potential dependence of nest hourly temperatures (Nest Th, C°, continuous) on the following variables fitted as fixed explanatory terms: Hourly ambient temperatures (AmbTh, C°, continuous), Nest type (Box, Hole, Tile), Nest orientation (categorical, two levels: Cold and Hot), as abiotic variables whereas Nestling number (numerical, 1-5) and Nestling age (categorical: a = 1-7, b = 8-14, c = 15-21, d = 22-28 days after hatching), as biotic variables. We attempted to include other weather variables (i.e. wind speed and rain precipitations) or other nest features (i.e. nest volume). All or part of these other potential explanatory variables, however, added too much variability to models so that their results (i.e. factor estimates) were meaningless. As a consequence we removed these variables from the model sets. Ambient temperature was recorded by the meteorological station on the Gela Plain (see above). Nest identity (i.e. ID, hereafter) nested in Colony ID and the Year (2010-2013) were fitted as random term of the models. Nests included in the analyses were the active ones, thus those cavities where at least one egg was laid. All active nests were included in the analysis from egg laying throughout incubation and until chick fledging or failure.

To further support our hypothesis, we compared the daily temperature means recorded among the three different nest types with an ANOVA.

#### Nest type occupancy

According to our results, kestrels should prefer nesting under tiles, thus the coolest nest type (see Results). As rooftops of the study colony buildings are increasingly collapsing (S1 Fig), we hypothesized tiles as a limited yet preferred resource that, as such, would be used by first breeders until available, then suboptimal nest types (i.e. holes) would be left to late conspecifics. In the colony buildings it is not possible to assess whether each roof tile is suitable as a nest and this makes it impracticable to estimate the total number of cavities available to lesser kestrels. Without a quantification of nest availability, we could not strictly test for nest preferences. We otherwise tested whether early breeders would preferentially occupy tiles by using one-way ANOVA where first Laying Day was the response variable and the nest type (two levels: Tile and Hole) the independent factor. We performed these analyses among nests in the colonies checked each of all nine study years. The ANOVA included all nests whose first laying day was known.

#### Nest temperature and reproductive success

We explored the effect of temperatures recorded inside the nests on nest success with the use of GLMMs. Seasonal decline of reproductive output, starting from smaller clutches, is a common trait especially among migratory species. Main causes of such a decline are decreased either resources or parent quality (date versus parent quality hypotheses). Late breeding attempts are also exposed to temperatures that increase naturally with the season. Consequently, seasonality may be a confounding factor when testing for the effects of nest temperature *per se* on nesting success. We accounted for this potential bias, by adjusting the reproductive success measures (i.e. number of hatchlings and fledglings) as the number of offspring produced by taking into account their natural reduction because of seasonality. We thus used the residuals of the number of hatchlings (r^2^ = 0.074, F_1,87_ = 6.93, P = 0.010) and fledglings (r^2^ = 0.141, F_1,81_ = 13.28, P < 0001) not explained by the first laying day as the two measures of reproductive performances. Thus, we obtained the residuals from two regressions, first, number of hatchlings versus the first laying day and, second, number of fledglings versus the first laying day.

Using thermologger records, we were able to quantify daily mean temperatures from hourly values. Nest temperatures were corrected by taking into account the influence of ambient temperatures. Thus, we defined the residuals of nest temperatures not explained by ambient temperatures (r^2^ = 0.237, F_1,87_ = 27.01, P < 0.0001) as nest microclimate. In one model, for each nest, microclimate mean recorded during incubation (i.e. from first day of laying to the day before hatching or nest failure) was used to test its predictive power on the residuals number of hatchlings. In another model, for each nest, nest microclimate mean recorded during chick rearing periods (i.e. from first day of hatching to fledging of the first chick or nest failure) was used to test its predictive power on the residuals number of fledglings. In addition to these three temperature values, we also included the Nest type (Box, Hole, Tile) as another fixed term in both models. In other words, we used two sets of GLMMs where the residuals of hatchlings and fledglings were our response variables and nest microclimate means and nest type the fixed terms. From the previous analyses resulted that nestlings affected significantly the nest temperatures. Accordingly, we fitted this term, the number of nestlings, as another random term in the second model set together with Nest ID nested in Colony ID. We adopted the same criteria described above for model selection and validation. To further support our hypothesis we tested the effect of nest temperatures of the proportion of hatchlings and nestlings. Specifically, we obtained the residuals of the proportion of hatchlings and fledglings by regressing them with the first laying day so to take into account a seasonality effect. Then we regressed the residuals of hatchlings and fledglings with the residuals of nest temperatures, as quantified above, by taking into account a potential effect of ambient temperatures.

At the University of Palermo there were no Institutional Animal Care and Use Committee or ethics committee so that approval was not necessary or required. Ringing was conducted as indicated by international ringing protocols, thus handling the birds the least necessary.

## Results

### Abiotic and biotic factors predicting nest temperatures

**Fig 1:**
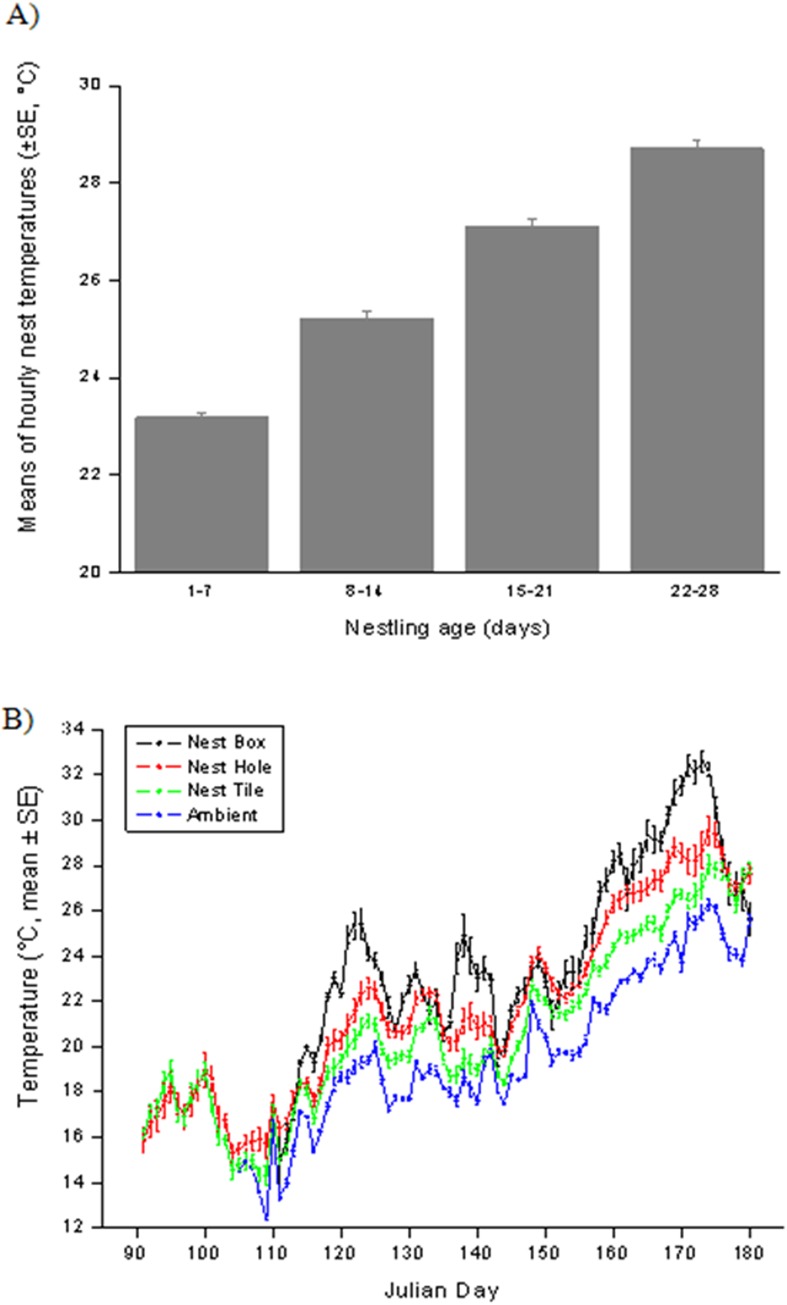
**Biotic (chick age, A) and abiotic (ambient temperature x nest type, B) factors as best predictors of nest temperatures as resulted from the best model selected.** As nestlings grew and acquired thermoregulatory capacities they also developed an increasing amount of heat that contributed to nest temperature increases (A). As the season became warmer, the different nest types diverged more and more in the in terms of their internal temperature, being nest boxes the warmest and nest tiles the coolest (B).

During the breeding season, nest temperatures were affected by ambient temperatures. The model including only this variable, however, is the poorest-performing one (Model no. 25 in Table 1) indicating that ambient temperature alone is insufficient in explaining variation in nest temperatures. The best model included, in addition to ambient temperature, nestling age, number of nestlings, nest type and an interaction between nestling age and number of nestlings (Table 1). Nests with a high number of chicks (Table 1) became warmer as chicks grew older (Fig 1A). For example, when daily ambient temperature mean was between 25 and 26°C, a nest with young chicks (age A and B) reached a daily mean of 27.9°C (n = 53) and, in comparison, nests with older chicks (age C and D, n = 96) reached a daily mean of 28.7°C. With the same ambient temperature, nests with 1-2 young chicks (age A) reached a temperature of 27.0°C (n = 5) whereas it was 30.3°C inside others with 5 old chicks (age D, n = 10). Boxes were the warmest nest types (daily hourly mean ± SE, 24.9 ± 0.1°C, n = 630), tiles the coolest (21.7 ± 0.1°C, n = 3036) and holes (22.7 ± 0.1°C, n = 2625) being of intermediate temperatures (Fig 1B).

**Table 1:** Full set of GLMMs testing for the effects of abiotic (weather metrics, nest features) and biotic (chick number and age) variables on temperatures recorded hourly inside 108 nests of lesser kestrels (191,035 temperature recordings in total). Model estimates are shown only for the best model.

Model no.	Model	Model Estimate	SE	AIC	ΔAIC	AIC weight	Number of parameters
**1**	AmbTh+Nstl_age+Nstl_no+Nest_type+Nstl_age*Nstl_no			**192348.50**	**0.00**	**0.69**	**13**
	AmbTh	0.611	0.00				
	Nstl_age_b	1.143	0.10				
	Nstl_age_c	1.217	0.10				
	Nstl_age_d	2.027	0.11				
	Nstl_no	0.043	0.03				
	Nest_type_hole	-0.388	0.45				
	Nest_type_tile	-2.358	0.46				
	Nstl_age b*Nstl_no	-0.021	0.03				
	Nstl_age c*Nstl_no	0.312	0.03				
	Nstl_age d*Nstl_no	0.294	0.04				
2	Amb_Th + Nstl_age + Nstls_no + Nest_type + Exposure + Nstl_age * Nstls_no			192350.40	1.90	0.27	14
3	Amb_Th + Nstl_age + Nstls_no + Nest_type + Exposure + Nstl_age * Nstls_no + Nest_type * Exposure			192353.90	5.40	0.05	16
4	Amb_Th + Nstl_age + Nstls_no + Exposure + Nstl_age * Nstls_no			192384.80	36.30	0.00	12
5	Amb_Th + Nstl_age + Nstls_no + Nstl_age * Nstls_no			192385.80	37.30	0.00	11
6	Amb_Th + Nstl_age + Nstls_no + Nest_type			192520.50	172.00	0.00	10
7	Amb_Th + Nstl_age + Nstls_no + Nest_type + Exposure			192522.40	173.90	0.00	11
8	Amb_Th + Nstl_age + Nstls_no + Nest_type + Exposure + Nest_type * Exposure			192526.00	177.50	0.00	13
Model no.	Model			AIC	ΔAIC	AIC weight	Number of parameters
9	Amb_Th + Nstl_age + Nest_type			192549.30	200.80	0.00	9
10	Amb_Th + Nstl_age + Nest_type + Exposure			192551.10	202.60	0.00	10
11	Amb_Th + Nstl_age + Nest_type + Exposure + Nest_type * Exposure			192554.40	205.90	0.00	12
12	Amb_Th + Nstl_age + Nstls_no + Exposure			192556.10	207.60	0.00	10
13	Amb_Th + Nstl_age + Nstls_no			192557.20	208.70	0.00	8
14	Amb_Th + Nstl_age + Exposure			192585.60	237.10	0.00	8
15	Amb_Th + Nstl_age			192587.20	238.70	0.00	7
16	Amb_Th + Nstls_no + Nest_type			196190.10	3841.60	0.00	7
17	Amb_Th + Nstls_no + Nest_type + Exposure			196191.10	3842.60	0.00	8
18	Amb_Th + Ntls_no + Nest_type + Exposure + Nest_type * Exposure			196191.90	3843.40	0.00	10
19	Amb_Th + Nstls_no + Exposure			196228.40	3879.90	0.00	6
20	Amb_Th + Nstls_no			196231.80	3883.30	0.00	5
21	Amb_Th + Nest_type			714760.00	522411.50	0.00	6
22	Amb_Th + Nest_type + Exposure			714761.40	522412.90	0.00	7
23	Amb_Th + Nest_type + Exposure + Nest_type * Exposure			714762.70	522414.20	0.00	9
24	Amb_Th + Exposure			714808.00	522459.50	0.00	5
25	Amb_Th			714811.90	522463.40	0.00	4

Amb_Th = hourly ambient temperature °C; Nstl_age = chick age, where reference category is Nstl_age_a (a = youngest, d = oldest); Nstl_no = number of chicks (1-5); Nest_type = type of nest, where reference category is Nest_type_box.

ANOVA results also supported our model result as nest temperatures were significantly different among the three nest types (ANOVA, F1,99 = 17.24, P < 0.00001), with tiles being the cooler (21.7 ± 0.2°C) than holes (23.1 ± 0.2°C, post-hoc Newman-Keuls test, P = 0.01) and boxes (24.9 ± 0.7°C, P = 0.0001) being these last warmer also than holes (P = 0.001).

### Nest type occupancy

**Fig 2:**
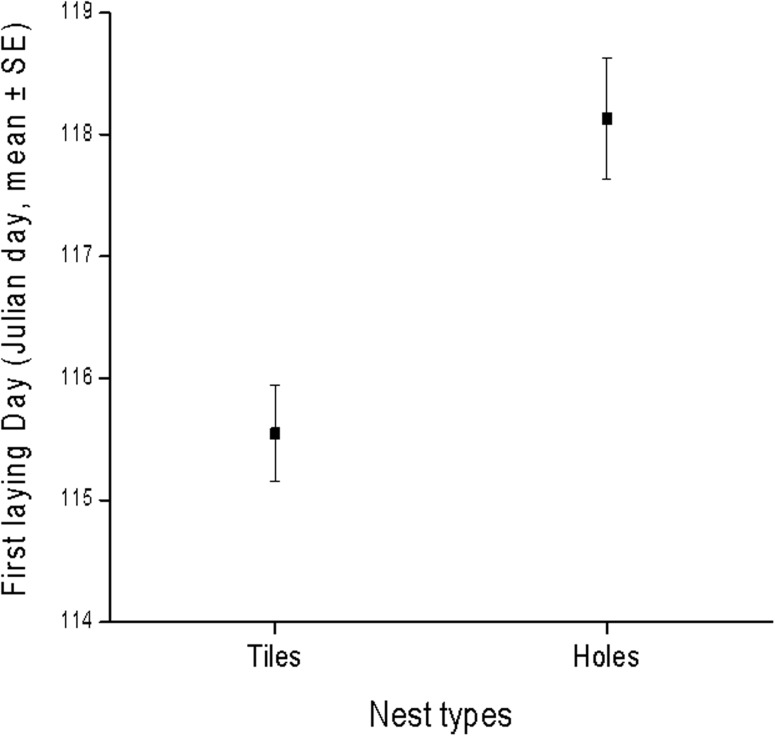
Nesting onset as expressed by the first laying day of 837 kestrel pairs nesting in 16 colonies during nine breeding seasons (2004-2013, with the exception of 2008).

A subsample of 837 nests in 16 colonies revealed pairs occupying tiles started laying significantly earlier (mean Julian day for onset of laying in tile nests was 115 ± 0.4 and in no-tile nests 118 ± 0.5; F_1,835_ = 17.0, p < 0.0001, Fig 2) than those breeding in holes, lending support to the idea that there is some preference for tile nests, among the nest types recorded, thus the nest type with the coolest insulation properties and not undergoing to overheating (Fig 1B).

### Nest temperature and reproductive success

**Fig 3:**
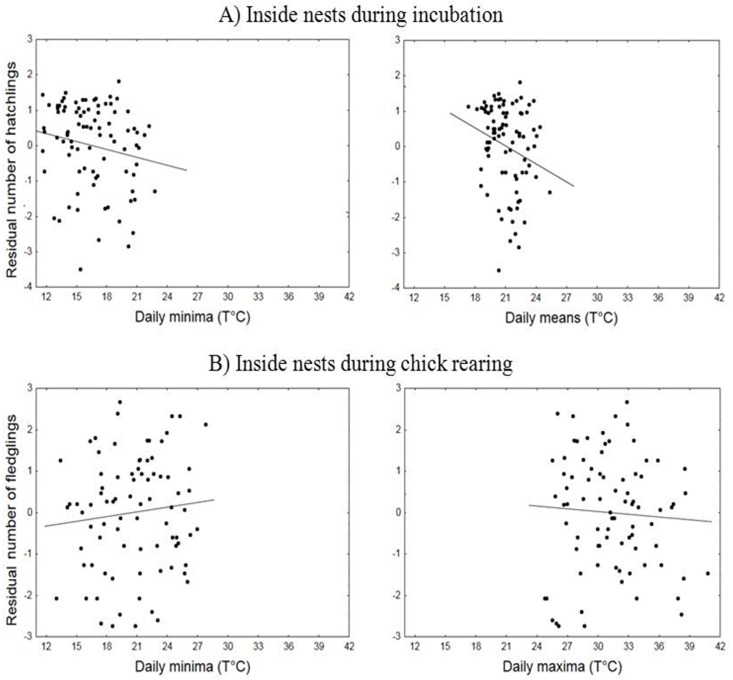
**Residuals not explained by first laying day and clutch size of the number of hatchlings (A) and fledglings (B) in 90 and 84 nests, respectively, of lesser kestrels as functions of daily temperatures.** Despite being the only predictor included in the best models, nest microclimate showed meaningless estimates indicating its not significant effect on nesting outcome. Lines show linear fits.

We could determine the number of hatchlings in 90 out of 100 nests equipped with thermologgers and, because of nest failure at the incubation stage, we determined the fledgling number on remaining 84 nests. The most important predictor of the residual number of hatchlings and fledglings was the daily mean of nest microclimate recorded during incubation and brooding stages, respectively (Table 2). Despite its effect in regulating the nest microclimate, the nest type was included in the models with the worse performances indicating its not significant effect on the breeding outcome. In both cases, despite some apparent trend, as visualized by the residuals of the number of nestlings not explained by laying day on the temperature metrics (Fig 3), temperature effects on reproductive output showed meaningless estimates so that each degree of nest microclimate resulted with 0.10 unhatched eggs and 0.06 more fledglings, regardless the first laying day (Table 2).

**Table 2:** GLMMs testing for the effect of potential predictors on the season-adjusted number of A) hatchlings and B) fledglings. Nest temperatures were recorded during A) incubation or B) brooding stages in 90 and 84 kestrel nests, respectively. Nest temperatures were corrected for the influence of ambient temperatures and defined as nest microclimate. Models with ΔAIC < 2 were selected as best models and shown in bold.

*A) Residual number of hatchlings*						
No.	Model	Model Estimate	SE	AIC	ΔAIC	Number of parameters
**1**	**Nest daily microclimate means (incubation period)**	**-0.109**	**0.10**	**306**	**0.0**	**4**
2	Nest Type			309	2.6	5
3	Nest daily means + Nest Type			310	3.1	6
4	Nest daily means + Nest Type + Nest daily means x Nest Type			312	5.6	8
5	Nest daily means x Nest Type			312	5.6	8
*B) Residual number of fledglings*						
No.	Model	Model Estimate	SE	AIC	ΔAIC	Number of parameters
**1**	**Nest daily microclimate means (brooding period)**	**0.061**	**0.06**	**295**	**0.0**	**5**
2	Nest Type			298	2.9	6
3	Nest daily means + Nest Type			299	4.0	7
4	Nest daily means x Nest Type			299	4.0	7
5	Nest daily means + Nest Type + Nest daily means x Nest Type			301	6.1	9

Supporting our model result, despite some apparent trend, hatchling (F_1,87_ = 0.18, R^2^ = 0.002 P = 0.674) and fledgling (F_1,81_ = 0.18, R^2^ = 0.01, P = 0.376) number did not show significant changes with nest temperatures.

## Discussion

We showed that our integrated analysis between micro- and macro-environmental factors is a useful tool to reveal crucial aspects to be taken into account when aiming to improve conservation actions applied to cavity nester species. First, we showed a clear dependence of nest temperature on biotic and abiotic factors. Nest temperatures were, not surprisingly, affected by ambient temperatures recorded at a macro-scale by the closest weather station. However, macro-habitat features on their own poorly predicted the micro-habitat conditions which were instead significantly estimated when also nest type and number and age of chicks were taken into account. The coolest thermal profile was provided by the roof tiles which were occupied by early breeders. On the contrary, later in the season, with much increased ambient temperatures, late breeders preferentially occupied the hottest type of nest. This is clearly consistent with tiles being a limited resource preferred by first breeders with late pairs occupying the remaining nest types, in line with the Ideal Pre-emptive Distribution idea by Pulliam and Danielson [26], according to which, the best territories are taken first and are then not available to others anymore. Following these findings, we expected to find tiles as the best nests hosting breeding attempts, thus providing the best microenvironmental conditions. In other words, according to our hypothesis, we expected to find better reproductive performances in tiles than in hotter nest types. Surprisingly, and on the contrary, tiles were not better sites than others, as breeding outcome was virtually equivalent in all types of nests. Other factors importantly affecting nest type selection, such as nest-type specific predatory pressure or public information [44], may better explain our findings.

Climate warming studies typically address the effects of temperature at a macro-scale level of observation [45, 46]. Physiological aspects, such as actual thresholds for heat stress, have been recently integrated into mechanistic models which have shown that including only ambient temperature in the models would ignore several biologically-relevant details [47]. Such details are important for addressing the call for urgently improving and integrating Species Distribution Models in providing reliable predictions for the future species distributions as global warming effects become more and more obvious [10]. Attempts to fill such gaps have focused on invertebrate organisms and aimed to downscale our predictions by quantifying thermal thresholds and examining, for example, physiological responses of automatons [48, 10] to local conditions. Consistently with similar studies, we show that environmental conditions at a micro-scale are crucial components in assessing the actual surroundings experienced by individuals and, for the first time, quantified and modelled the change of ambient temperature exposed to known biotic and abiotic factors until the resulting nest temperature.

Nest site selection is a behavioral trait promptly efficient to mitigate adverse overheating effects. Lesser kestrels are able to assess the different quality of nesting sites, as shown among Spanish breeders, preferentially selecting those colonies associated with the best trophic resources [49]. This species is however characterized by a despotic distribution with more experienced adults monopolizing the best resources at the expenses of young breeders who are displaced to the low-quality nest sites [49]. As mentioned above, although we could not perform a proper test for nest selection, our results are strongly consistent with a limitation of the cooler roof tiles, probably because of the gradual but constant collapsing of the unmaintained colony structures (S1 Fig). Based on our results plus former knowledge on this species and accepted climate predictions, kestrel populations would see ever increasing temperatures on their breeding site [50] that would result in wider thermal differences between nest types (this study) and with ever decreasing availabilities of proper nest types, especially for young breeders [49]. Ideally, the best conservation measure should aim to maintain colony roofs although this would involve nearly prohibitive costs associated. As suggested by other studies, although wooden boxes are the most available nests used for conservation purposes, they provide the worst conditions where high temperatures may get closer to lethal conditions (e.g. [19, 37, 51]. Creating new openings in already existing walls or building nests with the same material of tiles is an intuitive option but also more costly than the more common wooden boxes [15]. An interdisciplinary project, where material engineers, biologists and physiologists integrate their knowledge would help finding a both financially feasible and biologically efficient nest type offering insulation properties that are physiologically suitable to eggs, chicks and attending adults.

Thermal stress may reduce physiological performance or even survival when an individual faces temperatures above a specific threshold [24, 52, 53]. High temperatures induce damaging dehydration of both eggs and chicks [16, 53]. They can also trigger a detrimental cascade where parents first spend shorter time in attending nests, the absence of incubating parents then both increases the risk of egg microbial infections and predation, and decreases hatching synchrony potentially resulting in higher mortality of the youngest nestlings [54]. Accordingly, previous findings showed a decreased hatching and fledging success as temperature increased [15, 16].

Our results contrasted all the above findings. In Gela, hatching and fledgling success was unaffected not only by high ambient temperatures but also by the even higher temperatures found in poorly-insulated nests. One consistent explanation might be related to the ability of the breeding adults to keep temperatures far from lethal ranges for both eggs and chicks. In this case, adults, and not their breeding success, might have suffered from the excessive temperatures in overheated nests. Nest attendance is a costly activity that might be particularly requiring when thermoregulatory expenses become excessive and may involve medium- to long-term repercussions for the breeders [18]. This hypothesis might be verified by, on one side, recording temperatures within clutches and broods. This and similar investigations used thermologgers recording temperature inside nests but we do not know what is the screening power of the breeder body on clutches and broods. This investigation would also provide additional information on physiological threshold on egg exposure to high temperatures. Catry et al. [15, 37] depicted three possible scenarios on population growth depending on the adverse effect of different type of nests on chick conditions. This approach might be used by integrating the effect of different nest types also on adults into the kestrel population dynamics.

In a global scenario of warming temperatures, the coolest nest type increasingly missing from the breeding range would plausibly affect population trend. The fitness decline due to deteriorating habitat quality around the colonies recorded in the last ten years [39] can only be exacerbated by lack of optimal nest sites. Microevolution mechanisms may shift values of traits under selective pressure so that individuals maximize their fitness according to the changed environmental conditions [55, 56]. From our results also small clutches would have served to maintain cool temperatures inside nests. Reduction of clutch size is, however, a life-history trait not easily modified in a short time period [51], especially for raptors, but sensitive mostly on laying day [57].Other variables, such as nest attendance and nestling growth correlated with the distance of the colony from the foraging grounds may be more suitable to unveil breeding performances.

Future predictions of community distribution and structure entail facing the challenging task of addressing macro-scale phenomenon a with a micro-scale level of analysis. Lesser kestrel population in the Gela Plain has proved to be an ideal system to up-scale models from individual to population level. Future studies need to address more detailed both physiological response of eggs and chicks to nest overheating and availability quantification of differently-thermal nest sites in order to better improve current population management actions.

## Supporting information

S1 Data Nest temperatures and reproductive measures. (XLSX)

S2 Data Nest temperature statistics. (CSV)

S1 Fig **Examples of buildings hosting two lesser kestrel colonies in the Gela Plain (Italy) as in 2010 (A, B) and later in 2015 (C, D, respectively) and showing the progressive collapse of their rooftops.** We estimated (unpublished data) a decrease of 30-35% roof tiles available as potential nesting sites since the beginning of our study.
(DOCX)

S1 Table Description of the numbers of nests per colony and per year monitored during the study period. (DOCX)
