# Supplementary material for: An integrated analysis of micro- and macro-habitat features as a tool to detect weather-driven constraints: A case study with cavity nesters
Source: PLoS One. 2017 Mar 20;12(3):e0174090. doi: 10.1371/journal.pone.0174090 (PMC5358771; doi:10.1371/journal.pone.0174090)
Supplement: S1 Fig — Examples of buildings hosting two lesser kestrel colonies in the Gela Plain (Italy) as in 2010 (A, B) and later in 2015 (C, D, respectively) and showing the progressive collapse of their rooftops. We estimated (unpublished data) a decrease of 30-35% roof tiles available as potential nesting sites since the beginning of our study. (DOCX) [file pone.0174090.s003.docx]

**Supporting Information**

**An integrated analysis of micro- and macro-habitat features as a tool**

**to detect weather-driven constraints: a case study with cavity nesters**

D. Campobello^1,*^, J. Lindström ^2^, R. Di Maggio^1^, and M. Sarà^1^

^1^Section of Animal Biology, Dept. STEBICEF, Università di Palermo, 90123 Palermo, Italy

^2^ Institute of Biodiversity, Animal Health and Comparative Medicine, University of Glasgow, Glasgow G12 8QQ, UK

^*^ corresponding author: daniela.campobello@unipa.it; danielacampobello@hotmail.com

**S1Fig**. Examples of buildings hosting two lesser kestrel colonies in the Gela Plain (Italy) as in 2010 (A, B) and later in 2015 (C, D, respectively) and showing the progressive collapse of their rooftops. We estimated (unpublished data) a decrease of 30-35% roof tiles available as potential nesting sites since the beginning of our study.


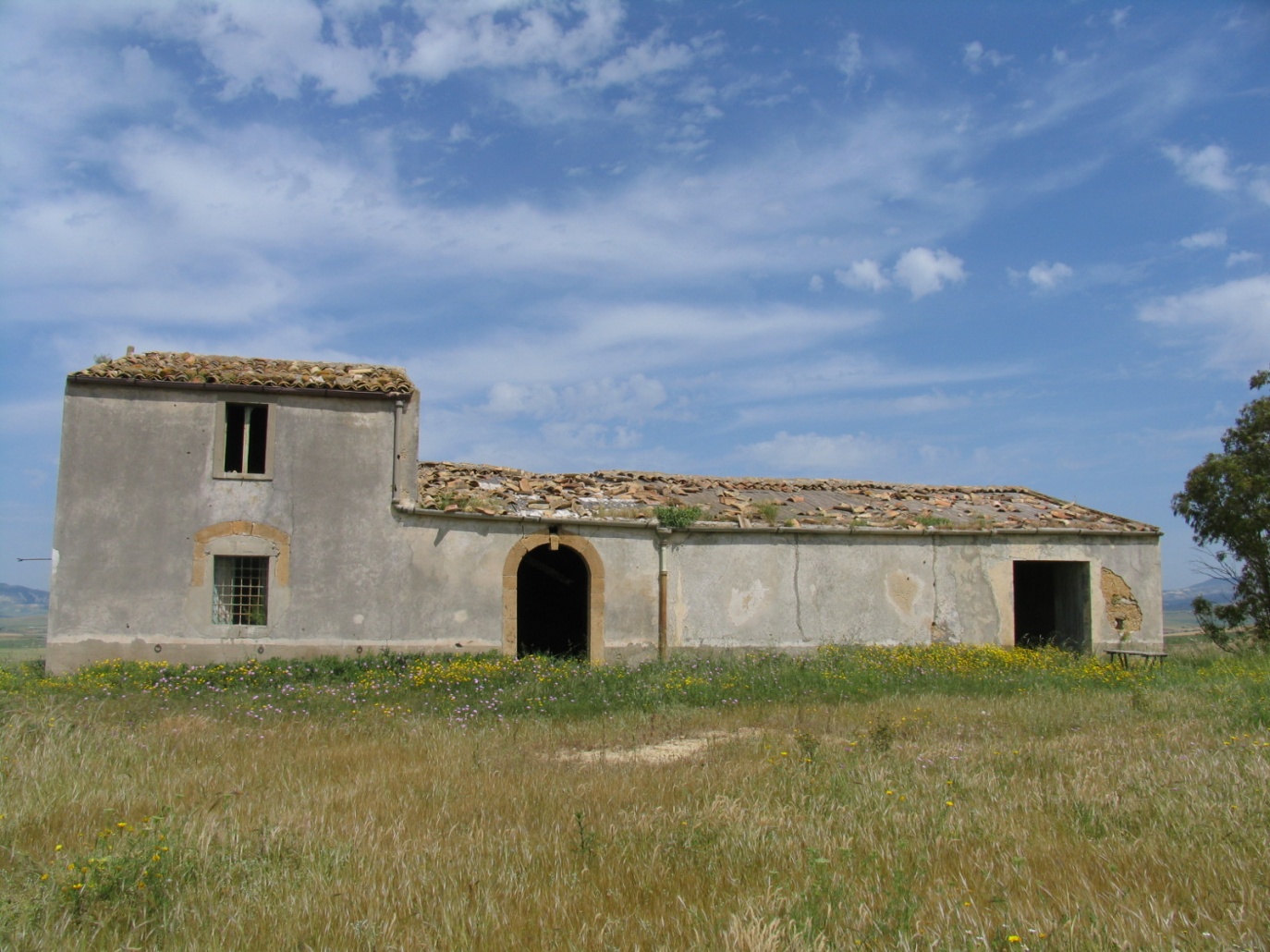


**A)**


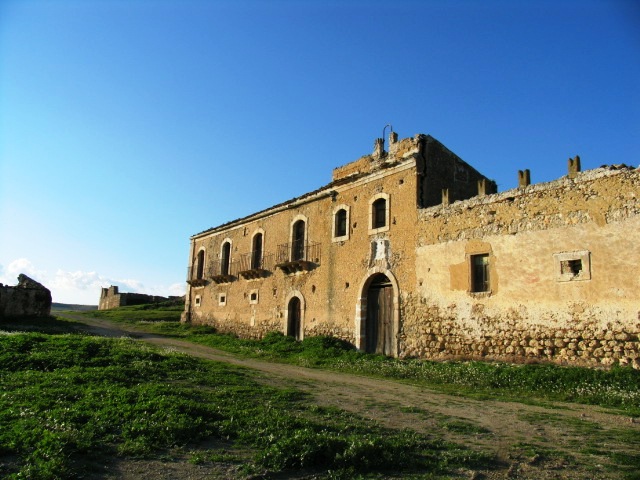


**B)**


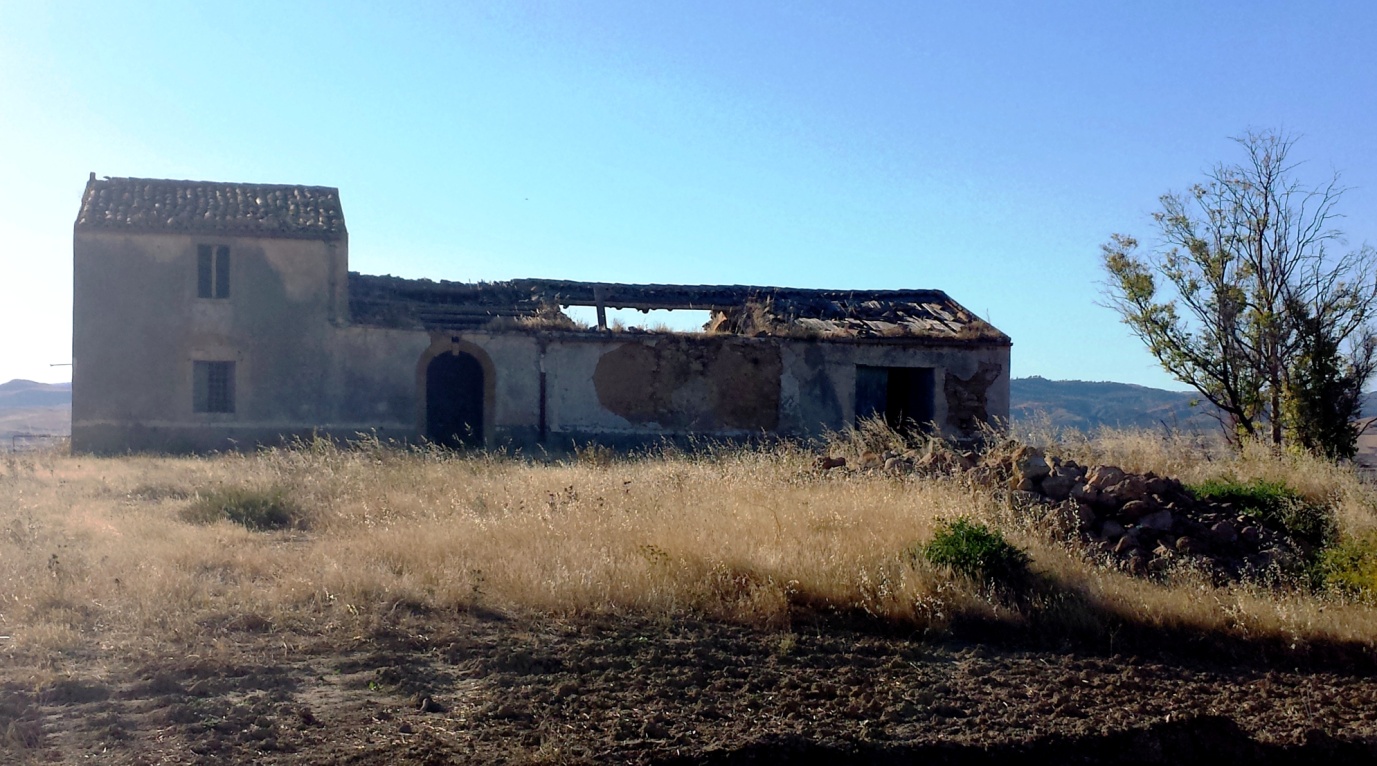


**C)**


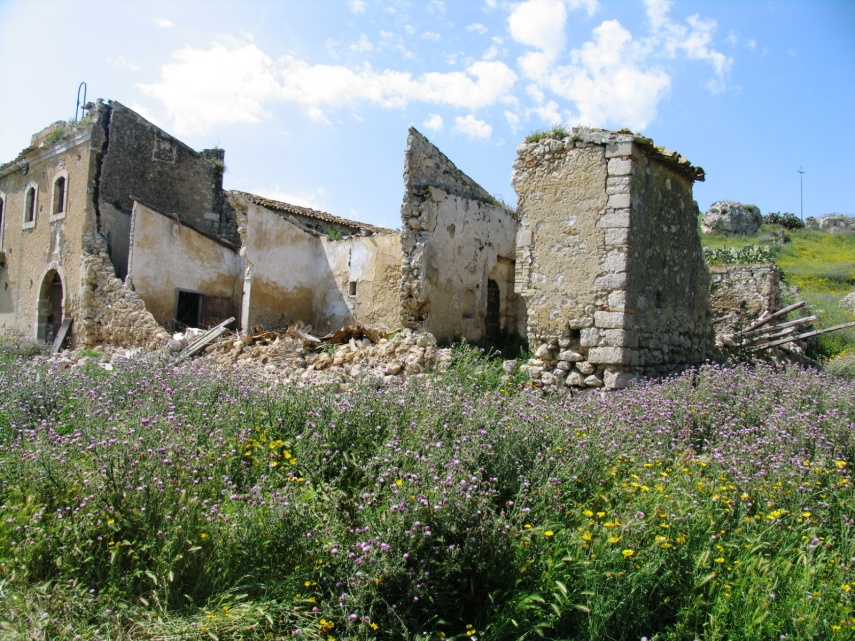


**D)**
