# Supplementary material for: An integrated analysis of micro- and macro-habitat features as a tool to detect weather-driven constraints: A case study with cavity nesters
Source: PLoS One. 2017 Mar 20;12(3):e0174090. doi: 10.1371/journal.pone.0174090 (PMC5358771; doi:10.1371/journal.pone.0174090)
Supplement: S1 Table — (DOCX) [file pone.0174090.s004.docx]

**Supporting Information**

**An integrated analysis of micro- and macro-habitat features as a tool**

**to detect weather-driven constraints: a case study with cavity nesters**

D. Campobello^1,*^, J. Lindström ^2^, R. Di Maggio^1^, and M. Sarà^1^

^1^Section of Animal Biology, Dept. STEBICEF, Università di Palermo, 90123 Palermo, Italy

^2^ Institute of Biodiversity, Animal Health and Comparative Medicine, University of Glasgow, Glasgow G12 8QQ, UK

^*^ corresponding author: daniela.campobello@unipa.it; danielacampobello@hotmail.com

**S1Table.** Full set of GLMMs showing the effects of potential predictors on the number of A) hatchlings and B) fledglings. Estimates of the models with ΔAIC <2 and predictor abbreviations are shown in table 2.

| **Model no.** | **Model** | **AIC** | **ΔAIC** | **AIC weight** | **Number of parameters** |
| --- | --- | --- | --- | --- | --- |
| *A) Number of hatchlings* | |  |  |  |  |
| 1 | LayD + CS | 319.8 | 0.0 | 0.185 | 4 |
| 2 | LayD + CS + Dmin | 320.4 | 0.6 | 0.139 | 5 |
| 3 | LayD + CS + Dmean | 320.5 | 0.7 | 0.128 | 5 |
| 4 | CS | 320.9 | 1.1 | 0.105 | 3 |
| 5 | LayD | 321.3 | 1.5 | 0.089 | 3 |
| 6 | Dmax + LayD + CS | 321.8 | 2.0 | 0.069 | 5 |
| 7 | Dmean | 321.8 | 2.0 | 0.068 | 3 |
| 8 | Dmean + Nest type + LayD + CS | 323.2 | 3.4 | 0.034 | 7 |
| 9 | Dmean + Nest type | 323.2 | 3.4 | 0.034 | 5 |
| 10 | Nest type + LayD + CS | 323.2 | 3.4 | 0.033 | 6 |
| 11 | Dmin | 323.3 | 3.5 | 0.032 | 3 |
| 12 | Dmin + Nest type + LayD + CS | 323.7 | 3.9 | 0.026 | 7 |
| 13 | Dmax + Nest type + LayD + CS | 325.0 | 5.2 | 0.014 | 7 |
| 14 | Dmean * Nest type | 325.8 | 6.0 | 0.009 | 7 |
| 15 | Dmin + Nest type | 326.1 | 6.3 | 0.008 | 5 |
| 16 | Dmean * Nest type + LayD + CS | 326.3 | 6.5 | 0.007 | 9 |
| 17 | Dmax | 326.7 | 6.9 | 0.006 | 3 |
| 18 | Nest type | 327.6 | 7.8 | 0.004 | 4 |
| 19 | Dmax + Nest type | 327.7 | 7.9 | 0.004 | 5 |
| 20 | Dmin * Nest type + LayD + CS | 327.7 | 7.9 | 0.004 | 9 |
| 21 | Dmax * Nest type + LayD + CS | 329.0 | 9.2 | 0.002 | 9 |
| 22 | Dmin * Nest type | 330.1 | 10.3 | 0.001 | 7 |
| 23 | Dmax * Nest type | 331.5 | 11.7 | 0.001 | 7 |
| 24 | Dmean + Dmin + Dmax + Nest type+ Dmean * Nest type + Dmin * Nest type +Dmax * Nest type +LayD + CS | 336.5 | 16.7 | 0.000 | 15 |
| **Model no.** | **Model** | **AIC** | **ΔAIC** | **AIC weight** | **Number of parameters** |
| *B) Number of fledglings* | |  |  |  |  |
| **1** | **LayD + CS** | **300.5** | **0.0** | **0.333** | **4** |
| **2** | **Dmin + LayD + CS** | **302.0** | **1.5** | **0.157** | **5** |
| **3** | **Dmax + LayD + CS** | **302.3** | **1.7** | **0.140** | **5** |
| 4 | Dmean + LayD + CS | 302.5 | 2.0 | 0.123 | 5 |
| 5 | LayD | 302.6 | 2.0 | 0.120 | 3 |
| 6 | Nest type + LayD + CS | 304.2 | 3.7 | 0.053 | 6 |
| 7 | Dmin + Nest type + LayD + CS | 305.7 | 5.1 | 0.026 | 7 |
| 8 | Dmax + Nest type + LayD + CS | 305.7 | 5.1 | 0.026 | 7 |
| 9 | Dmean + Nest type + LayD + CS | 306.1 | 5.5 | 0.021 | 7 |
| 10 | CS | 306.6 | 6.0 | 0.016 | 3 |
| 11 | Dmax*Nest type + LayD + CS | 308.6 | 8.1 | 0.006 | 9 |
| 12 | Dmin*Nest type + LayD + CS | 309.3 | 8.7 | 0.004 | 9 |
| 13 | Dmean*Nest type + LayD + CS | 309.7 | 9.1 | 0.003 | 9 |
| 14 | Dmax | 311.9 | 11.4 | 0.001 | 3 |
| 15 | Dmean | 312.4 | 11.9 | 0.001 | 3 |
| 16 | Dmean + Dmin + Dmax + Nest type +Dmean*Nest type + Dmin*Nest type + Dmax*Nest type + LayD + CS | 312.7 | 12.1 | 0.001 | 15 |
| 17 | Dmin | 313.7 | 13.1 | 0.000 | 3 |
| 18 | Dmax + Nest type | 314.3 | 13.8 | 0.000 | 5 |
| 19 | Nest type | 315.2 | 14.7 | 0.000 | 4 |
| 20 | Dmean + Nest type | 315.5 | 15.0 | 0.000 | 5 |
| 21 | Dmin + Nest type | 317.2 | 16.6 | 0.000 | 5 |
| 22 | Dmax*Nest type | 317.4 | 16.9 | 0.000 | 7 |
| 23 | Dmean*Nest type | 318.6 | 18.0 | 0.000 | 7 |
| 24 | Dmin*Nest type | 320.8 | 20.3 | 0.000 | 7 |
| Total |  |  |  | 1.000 |  |
